# A bidirectional shift: how DNA methylation dynamics protect polyploid rice from heat stress

**DOI:** 10.1093/plphys/kiag209

**Published:** 2026-04-13

**Authors:** Guannan Wang

**Affiliations:** Assistant Features Editor, Plant Physiology, American Society of Plant Biologists, Rockville, MD 20855, United States; Department of Biology, Stanford University, Stanford, CA 94305, United States; Howard Hughes Medical Institute, Stanford University, Stanford, CA 94305, United States

Polyploidy, the condition of possessing 3 or more complete sets of chromosomes, serves as a major evolutionary mechanism that significantly enhances a plant's adaptability to environmental challenges compared to their diploid progenitors and relatives (Van de Peer et al. 2021). When confronting abiotic stresses such as drought, salinity, and heat, polyploid plants generally outperform diploids by employing structural and physiological advantages, including thicker cuticles, deeper root systems, reduced stomatal density to minimize water loss, and a more robust antioxidant defense system to mitigate cellular damage (Tossi et al. 2022). Similarly, polyploidization also provides distinct benefits against biotic threats. These plants frequently exhibit enhanced resistance to pathogens and herbivorous pests due to increased allelic diversity and a boosted production of protective secondary metabolites, such as alkaloids and flavonoids (Tossi et al. 2022).

As one of the major environmental challenges, heat stress negatively impacts the growth and development of plants and ultimately often causes cell damage and death. As a consequence, heat stress has led to significant reductions in global crop yield and poses a major threat to agricultural sustainability and food security, especially during an era when frequent extremely high temperatures are becoming the new norm as a result of global warming. Modeling has predicted that every 1 ℃ increase in temperatures reduces the yield of major crops by 3% to 7% (Zhao et al. 2017). As global temperature continues to rise, enhancing the resilience of plants, particularly crops, against heat stress is critical for ensuring food security. While extensive progress has been made on the understanding of plant responses to heat stress, the question of how polyploidization shapes heat stress responses in rice, the primary stable food for more than half of the world's population (Muthayya et al. 2014), remains largely unexplored. In this issue of *Plant Physiology*, Zhang and colleagues (Zhang et al. 2026) investigated the link between ploidy levels and heat stress responses by comparing the heat responses of 2 rice cytotypes, diploid japonica rice (GFD-2X) and its corresponding autotetraploid cytotype (GFD-4X), through an integrated analysis of physiological, transcriptomic, and epigenetic changes.

To elicit heat stress in rice with different ploidy levels, the authors treated both GFD-2X and GFD-4X rice lines at 40 ℃ for 7 days, and subsequently recovered treated plants under normal conditions (26 ℃ and 20 ℃ for day and night respectively) for another 7 days. The GFD-4X originated from the natural doubling of GFD-2X during cultivation and has been self-pollinated for generations (Leng et al. 2023). Compared to GFD-2X, the GFD-4X plants exhibited less pronounced leaf rolling, wilting, and chlorosis, and milder reductions in critical growth metrics, such as fresh and dry weights and shoot and root lengths, during both the treatment and recovery phases. Consistently, GFD-4X maintained a significantly higher relative water content and a lower overall water loss rate than GFD-2X. While transpiration rates declined in both cytotypes, the reduction was more pronounced in GFD-4X (20%) than GFD-2X (13%). Additionally, GFD-4X displayed a stronger reactive oxygen species (ROS) scavenging capacity as heat-induced accumulation of superoxide radicals and hydrogen peroxide was much lower in GFD-4X than GFD-2X lines. All quantified antioxidant indicators, including antioxidative enzymes, soluble sugars, malondialdehyde (MDA), and proline, exhibited a stronger induction upon heat stress in GFD-4X than GFD-2X. These findings together suggest that the tetraploid rice exhibits enhanced physiological resilience to heat stress compared to its diploid counterpart.

As previous studies have repeatedly shown that plant hormones are central for plant stress responses (Waadt et al. 2022), the authors tracked the changes in indole-3-acetic acid (IAA), indole-3-butyric acid (IBA), ethylene (ETH), abscisic acid (ABA), salicylic acid (SA), jasmonic acid (JA), trans-zeatin (tZ), and gibberellin (GA) in both diploid and tetraploid rice plants during both heat stress and recovery phases. Intriguingly, the content of all hormones increased significantly in both GFD-2X and GFD-4X plants after heat treatment, and GFD-4X consistently exhibited higher concentrations of all hormones than GFD-2X. The hormone levels decreased during the recovery phase, but most were still higher than their respective control plants for both cytotypes, particularly for GFD-4X.

The overall transcriptomic profiles remained largely similar between GFD-2X and GFD-4X before and after heat stress, despite the fact that hundreds of genes exhibited ploidy-dependent expression. GFD-4X plants exhibited a broader transcriptional response to heat stress, with over 2,000 more heat-responsive genes than GFD-2X plants. Over 50% of the heat-responsive genes in GFD-2X were also found differentially regulated in GFD-4X under elevated temperature. This proportion increased to nearly 70% during the recovery phase, indicating a highly shared core heat response and recovery program between the diploid and the tetraploid rice. Among these shared heat-response genes between GFD-2X and GFD-4X, processes related to RNA modifications, heat responses, and responses to hydrogen peroxide were highly enriched. Similar processes were also found overrepresented among genes that were only induced by heat in GFD-4X plants, suggesting that the GFD-4X plants mount an expanded and more comprehensive response to heat stress on top of the core responsive programs, potentially providing additional protection and enhancing plant tolerance to elevated temperatures.

The authors further compared genome-wide DNA methylation profiles between the diploid and tetraploid rice plants. During both heat stress and recovery phases, GFD-4X consistently possessed higher global DNA methylation levels across CG, CHG, and CHH contexts, indicating that the act of polyploidization in rice itself increases DNA methylation. Heat stress triggered a global decrease in DNA methylation across all sequence contexts in both cytotypes, with GFD-4X showing approximately 12% less reduction compared to GFD-2X. However, because GFD-4X possesses a much higher baseline of methylation, the smaller relative percentage drop could represent a highly targeted stripping of suppressive marks from specific functional regions. As evidence of this, GFD-4X generated more than 2 thousand hypomethylated regions (hypo-DMRs), whereas the diploid GFD-2X only managed to generate hundreds of hypo-DMRs. GFD-4X exhibited severe demethylation in critical functional areas, with promoter methylation decreasing more sharply than in GFD-2X, and gene body methylation—which was initially higher—plunging below diploid levels (Fig. 1). Further detailed analyses revealed that this massive targeted demethylation was well correlated with the rapid activation of genes regulating hormone biosynthesis and signaling, ROS-scavenging peroxisomes, and heat shock proteins (such as HSP20, HSP90B), thereby enabling a superior survival response.

**Figure 1:**
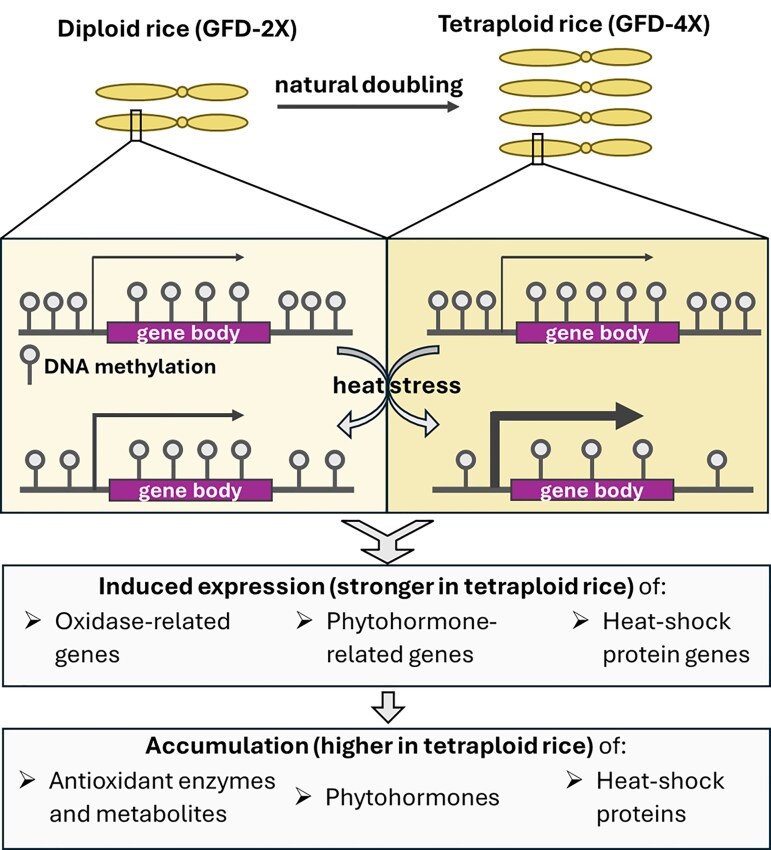
Heat stress responses of diploid (GFD-2X) and tetraploid (GFD-4X) rice plants. The visualization was modified from Zhang et al. (2026).

The work by Zhang and colleagues (Zhang et al. 2026) revealed that autotetraploid rice (GFD-4X) exhibits significantly greater heat tolerance than its diploid counterpart (GFD-2X) due to superior water retention, ROS scavenging, and a more robust transcriptomic response. This advantage is driven by a dynamic “bidirectional shift” in the epigenome: polyploidization naturally induces widespread DNA hypermethylation under normal conditions, but acute heat stress triggers a massive, highly targeted hypomethylation event. This rapid removal of suppressive epigenetic marks acts as a release mechanism, allowing the tetraploid plant to activate key survival genes responsible for producing protective hormones, antioxidant enzymes, and heat shock proteins (Fig. 1). Looking forward, it will be interesting to investigate what are the molecular switches mediating the differential heat responses in the tetraploid rice, how the tetraploid rice responds to long-term heat stress and compound stresses that often occur in field conditions, how the tetraploid balances the trade-off between enhanced stress response and growth, and what are the essential regulatory modules that will be needed to engineer climate-ready rice varieties. Ultimately, deciphering this complex epigenetic and transcriptomic network and its implications in field-ready solutions for crop improvement may provide the foundational blueprint required to protect global agricultural productivity against escalating climate extremes.

## Recent related articles in *Plant Physiology:*

Yu et al. (2025) compared the single-based resolution DNA methylation between the cultivated allotetraploid peanut (*Arachis hypogaea*) and its ancestral diploid parents (*Arachis duranensis* and *Arachis ipaensis*).

López-Jurado et al. (2025) investigated the relationship between ploidy levels and whole-plant water relations using different ploidies (2x, 4x, 6x, and 12x) of *Dianthus broteri*.

## Data Availability

No new data were generated or analyzed in support of this research.
